# Isolation of Phycobiliproteins from *Thermosynechococcus* PCC 6715 by Foam Fractionation in Batch and Continuous Modes

**DOI:** 10.3390/md24010033

**Published:** 2026-01-09

**Authors:** Anna Antecka, Rafał Szeląg, Stanisław Ledakowicz

**Affiliations:** Department of Bioprocess Engineering, Faculty of Process and Environmental Engineering, Lodz University of Technology, 213 Wolczanska Street, 93-005 Lodz, Poland; rafal.szelag27@gmail.com (R.S.); stanislaw.ledakowicz@p.lodz.pl (S.L.)

**Keywords:** phycobiliproteins, phycocyanin, allophycocyanin, isolation, foam fractionation

## Abstract

Phycobiliproteins are recognized as potential bioactive compounds and described as highly valued natural products for industrial and biotechnological applications. Moreover, they have been observed to possess antioxidant, anticancer/antineoplastic, and anti-inflammatory activities. Therefore, the search for new methods of their extraction and isolation is still ongoing. Foam fractionation, a bubble separation technique that allows amphiphilic molecules to be separated from their aqueous solutions, is a promising but understudied method. The process may be carried out both under mild conditions that are suitable for proteins and also for diluted solutions. This paper presents the results of applying the foam fractionation process to concentrate and separate phycobiliproteins. Allo- and C-phycocyanin from a thermophilic *Synechococcus* PCC 6715 strain were used in extract form after biomass cultivation and disintegration. Two ways of running the process were investigated: batch mode and continuous mode, the latter of which has not been reported in the literature previously. The results indicate that the method can be applied on a larger scale, as the outcomes of the continuous mode processes were comparable to those of the batch mode. Moreover, the results indicate that the process provides, to a certain extent, the opportunity of separating phycobiliproteins from each other.

## 1. Introduction

Phycobiliproteins (PBPs) are a group of colored, water-soluble proteins with a covalently attached tetrapyrrole chromophore, responsible for the light-harvesting in photosynthesis, produced by cyanobacteria and certain algae. Their primary role is to capture light energy across a wide range of wavelengths. They are organized into large structures called phycobilisomes, which are attached to the thylakoid membrane. These proteins, which include phycocyanin (C-PC), allophycocyanin (APC), and phycoerythrin (PE), absorb light energy and transfer it to chlorophyll. In cyanobacteria the most common phycobiliprotein is phycocyanin, whose absorption maxima is approximately 620 nm. This protein is composed of two α and β subunits with a hexameric conformation (αβ)6 at pH 5.0–6.0 and a trimeric conformation (αβ)3 at pH 7.0 [1]. Phycocyanin is recognized by radical scavenging activity, which was already verified in vitro. It can scavenge various reactive oxygen species such as superoxide, hydroxyl radical, the hydrogen peroxide radical peroxyl group, and the hypochlorous acid molecule. Moreover, it has been discovered that this blue pigment was not only able to react with hydroxyl, alkoxyl, and peroxyl radical but also able to react with peroxynitrite (ONOO-) and hypochlorous acid (HOCL) [2]. Phycocyanin is also reported for its various pharmacological properties other than antioxidant activity such as anti-inflammatory, neuroprotective, and hepatoprotective properties. These properties have been correlated to phycocyanin in various experiments [3,4]. Because of its aforementioned non-specific properties, C-PC is of great importance as a potential bioactive compound for industrial applications. It can be employed as a natural colorant in food, cosmetics, and pharmaceuticals; in pharmaceutical settings, it can be used to help cope with anemia and liver disease, and it is regarded as a nutraceutical which strengthens the immune system and has anticancer properties (interestingly it appears to be toxic to cancer/neoplastic cells without exerting any toxicity to normal/healthy cells [3,4]).

However, the prices of phycobiliproteins have recently reached very high levels, ranging from 3000 to 25,000 USD/kg depending on purity [5]. Therefore, downstream processing (DSP), which accounts for as much as 50–80% of the cost of the production of bioproducts [6], appears to be a key step worth exploring and optimizing for a more sustainable production process. The classical methods used for concentration and purification of PBPs, such as ultrafiltration and chromatography, are effective but very expensive and time-consuming. Therefore, the development and utilization of new approaches is still of great interest. A promising, yet understudied method is foam fractionation (FF) [7]. It is a bubble separation technique, in which hydrophobic molecules are preferentially separated from a liquid solution using rising columns of foam [8]. During the process a liquid phase is fed with dispersed gas stream and forms a foam phase which contains the particles and chemical compounds with affinity to the gas–liquid interface [9]. The collected bubbles collapse, forming a new liquid phase containing concentrated product. Despite the general simplicity, there are a number of factors influencing the effectiveness of foam separation which result in different values of enrichment and purification factors. Firstly, various feed and gas flow rates affect the nature of the bubbles such as their shape and size [10]. Secondly, high protein affinity towards the gas–liquid interface highly depends on the pH value of the initial liquid phase. The first application of the method for protein purification was conducted in 1940 by Ostwald and Mischke for the separation of products from yeast fermentation broth [11]. Later on, the method was widely used for the removal of detergents, metal ions, and solvents from water phases [12]. Nowadays, the technology has been employed in wastewater treatment, remediation, metallurgy, biotechnology, pharmacy, and the cosmetics and food industries [13]. Primarily, in all applications it is reported as an environment-friendly and sustainable DSP technology [14], since it does not require solvents or high-energy efforts. Another advantage is that it can be easily performed with a continuous supply of feed and receive solution of foamate and retentate [9].

Our earlier studies [15,16] confirmed the validity of using the foam fractionation method to separate phycobiliproteins. However, this method was only investigated for phycocyanin separation in batch mode. Meanwhile, continuous biological production is increasingly recognized as a means of intensifying biopharmaceutical production, particularly in downstream processing [17]. Using continuous operations reduces production costs, including capital outlay and running costs, by reducing the space occupied by equipment and increasing its utilization. The characteristics of integrated continuous manufacturing processes, the requirements needed to implement them, and the results achieved through implementation and scaling out are presented by Jungbauer et al. [18].

Therefore, the main aim of this research was to explore the continuous process, which, to the best of our knowledge, is entirely novel to the literature. Moreover, this study describes the purity and recovery of phycocyanin and allophycocyanin, as well as the possibility of separating the two from each other. This study presents new, improved results regarding the purity of PBPs, the recovery yield of PBPs in condensates and retentates, and the enrichment and partitioning coefficients for C-PC in both the batch and continuous modes.

## 2. Results and Discussion

### 2.1. Batch Foam Fractionation

In the FF process, the stream of gas introduced into the protein solution causes bubbles to form. At the same time, the proteins contained in the solution diffuse to the gas–liquid interface and, as a result of adsorption at this interface, are carried up the column together with the foam [19]. Therefore, an important parameter to be tested is the air flow rate, which should ensure the smallest possible volume of foam condensate with the highest possible degree of C-PC purity. For a given column with specific geometry and based on previous experiments conducted at the Department of Bioprocess Engineering [15,16,20], flow rates in the range of 2.4–6.0 L/h were selected and further investigated. The experiments were carried out for four different values of air flow rates: 2.4, 3.5, 4.8, and 6.0 L/h. Below 2.4 L/h, the amount of foam produced was insufficient to reach the top of the column, while above 6.0 L/h, excessive foaming occurred and a significant amount of liquid flowed along with the foam to the condensate. All processes were conducted at a room temperature of 25 °C and native pH of approximately 7.5. These conditions are neutral towards phycobiliproteins and ensured a gentle and effective process. In an FF process, too high pH causes the decomposition of PBPs, which is visible as a loss of color and excessive foaming, while a low pH, below the isoelectric point, contributes to the precipitation of the crude extract. In the case of temperature, lower values decrease the process yields, while at temperatures above 50 °C, the crude extract undergoes partial decomposition.

In the table (Table 1) the average volumes of foamates and retentates obtained after the FF processes as well as the degree of volume reduction are presented.

**Figure 1:**
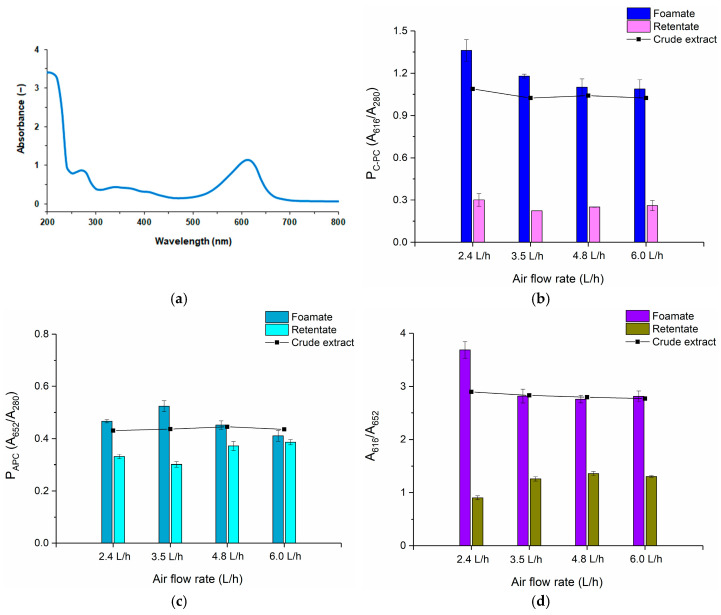
(**a**) Absorption spectrum of crude extract of phycobiliproteins (PBPs), (**b**) purity of C-phycocyanin (C-PC), (**c**) purity of allophycocyanin (APC), (**d**) A_616_/A_652_ in individual fractions in batch foam fractionation for different air flow rates.

The experimental results show that with the higher air flow rate, the volume of foamate collected grew, so the degree of volume reduction decreased and, at the same time, the concentration of C-PC in the foamate decreased. Almost sevenfold volume reduction with the volume of concentrate of 18 mL at a flow rate of 2.4 L/h was observed, while at an air flow rate of 6.0 L/h, the reduction was only 1.5 and the amount of foam condensate increased to 67 mL, and it was visibly diluted. The highest C-PC concentration in the foamate, equal to 1.15 mg/mL, was obtained at an air flow rate of 2.4 L/h, with an initial C-PC concentration in the crude extract of 0.28 mg/mL. In Figure 1a the absorption spectrum of the crude extract of PBPs is presented, from which C-PC was identified in the wavelength of 616 nm and APC of 652 nm.

**Figure 2:**
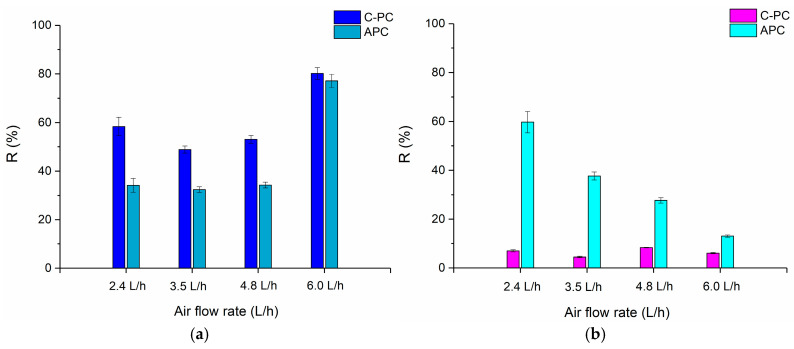
The recoveries of phycobiliproteins in (**a**) foamates and (**b**) retentates for different air flow rates.

Foam fractionation of the crude extract resulted not only in the concentration of C-phycocyanin but also its partial purification. The highest purity of C-PC was obtained at an air flow rate of 2.4 L/h and amounted to approximately 1.36, with a purification factor of 1.25 (Figure 1b). For higher flow rates, the purity of C-PC in the foam condensates was only slightly higher than in the crude extracts. This indicates that some other proteins, including allophycocyanin, were also transferred with the foam to the condensate. As can be seen from the graph (Figure 1c), APC was present in both the foam condensate and the retentate; however, its purity in retentates was higher in every experiment than for the purity of C-PC. The observed phenomena will be more visible when the recovery ratio of PBPs is considered. The scores presented in Figure 1d confirm that the most efficient C-PC separation from APC occurred when the air flow rate was equal to 2.4 L/h. In Figure 2 the recoveries of two analyzed PBPs both in the foamates and retentates obtained after the processes are shown.

The average recovery of C-PC in the foamate for air flow rates between 2.4 and 4.8 L/h was 50% (Figure 2a), while that of APC was approximately 30%. The highest C-PC recovery value, approximately 80%, was recorded at the highest air flow rate of 6.0 L/h, with similar values obtained for APC. This was due to the large volume of foam condensate obtained as a result of intense foaming of the raw extract solution. The C-PC residue in the retentate did not exceed 10%, regardless of the set air flow rate (Figure 2b), in contrast to APC, where its recovery increased with decreasing air flow rate—the highest, almost 60%, was observed at a flow rate of 2.4 L/h. For flow rates of 2.4–4.8 L/h, the separation of two phycobiliproteins between the foamate and retentate phases was observed. This means that C-PC diffuses to the phase boundary in greater quantities than APC and rises with the foam up the column, eventually forming condensate. In contrast, more APC remains in the retentate. This is particularly evident at lower air flow rates and confirms the observation that C-PC purity in foam condensate is the highest at an air flow rate of 2.4 L/h.

**Figure 3:**
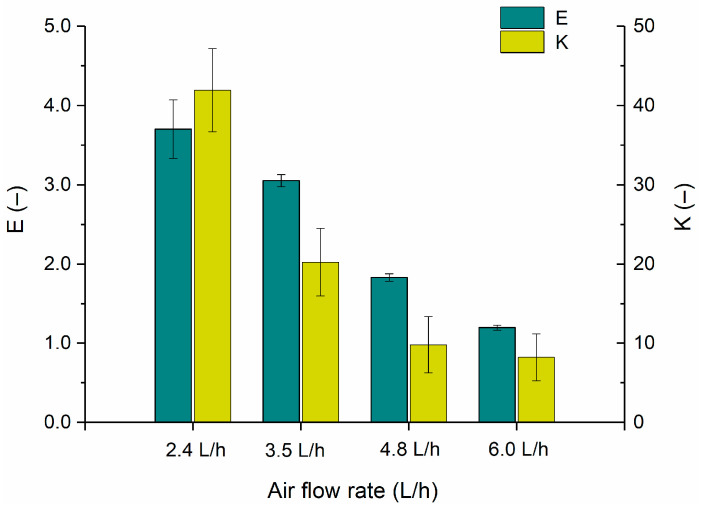
The foam enrichment coefficient E (C_C-PC_ in the foamate/C_C-PC_ in crude extract) and partitioning coefficient K (C_C-PC_ in the foamate/C_C-PC_ in the retentate) of C-phycocyanin for different air flow rates.

The following graph (Figure 3) shows the values of foam enrichment and partitioning coefficients obtained for the C-PC in the processes discussed.

The values of the foam enrichment coefficients and partitioning coefficients obtained (Figure 3) were directly proportional to the C-PC concentrations achieved after fractionation. At a flow rate of 2.4 L/h, the highest foam enrichment coefficient of 3.7 was obtained, while at 6.0 L/h it was only 1.2. The partitioning coefficient at an air flow rate of 2.4 L/h was 42, which was four times higher than at flow rates of 4.8 and 6.0 L/h. This indicates greater C-PC concentration efficiency at lower air flow rates. This was also confirmed by the literature data [9], which shows that at lower values of the gas flow rate supplied to the column, the gas–liquid contact time increases, smaller bubbles are formed, and the residence time of the foam in the column is extended, resulting in its drying under the influence of gravitational liquid fall. At higher air flow rates, the amount of liquid transferred to the foam condensate increases and the foam is less enriched.

### 2.2. Continuous Foam Fractionation

The results presented for the experiments performed in batch mode were the basis for comparing and contrasting the scores from the continuous processes. The design of the column used before for the batch mode enabled continuous processing through the connection of two peristaltic pumps; one ensured a constant supply of crude extract (substrate) and the second the continuous removal of excess retentate. In this mode the air flow rate and the streams of phycocyanin extract and retentate must be carefully selected to ensure the incoming phycocyanin extract has a sufficiently long residence time in the column to allow it to foam. The experiments were conducted at three different values of air flow rates, 2.4, 3.6, and 4.8 L/h, and two experimentally determined flow rates of crude extract flowing into the column and retentate collected during the process, set at 2 and 4 mL/min. This resulted in six variants of the experiment, the parameters of which are presented in the table (Table 2).

For each tested air flow rate, both the values of the supplied extract and the collected retentate flow rates were investigated. An air flow rate of 2.4 L/h, despite the favorable results obtained for the batch mode, proved insufficient to maintain the process in continuous mode. For this reason, all the results presented below are for processes 3–6 (Table 2).

**Figure 4:**
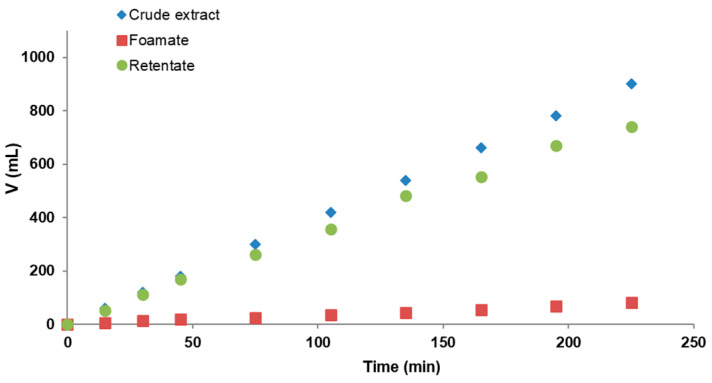
Volumes of individual streams in the continuous foam fractionation for process no. 6 (air flow rate of 4.8 L/h and crude extract and retentate flow rates of 4 mL/min).

In the following processes there were two kinds of products: foamate collected periodically in the form of foam condensate and retentate. Therefore, the total volume of them should be equal to the volume of crude extract supplied to the column. In reality, due to the losses of solution in the column, this was not possible to achieve. However, the observed losses were rather low and constant, so that the proportions of the quantities of the products obtained in relation to the crude extract remained stable. The residence time was 25 min for the flow rates of extract and retentate of 4 mL/min and was extended to 50 min for flow rates of 2 mL/min. In the following graph (Figure 4) the three discussed streams of crude extract, foamate, and retentate for experiment no. 6 (air flow rate of 4.8 L/h and pump flow rates of 4 mL/min) are presented.

Similar results to experiment 6 (Figure 4) were obtained for the other three processes. The volumes of foam condensates obtained were significantly lower than those for the retentates collected, indicating effective PBP concentration. Process stabilizations were achieved approximately 100 min after their initiation.

**Figure 5:**
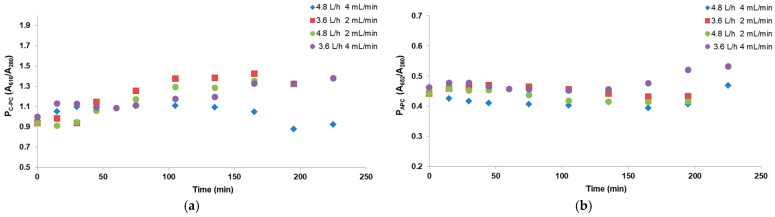
Purity of (**a**) C-phycocyanin in the foamates and (**b**) allophycocyanin in the retentates after continuous foam fractionation for the 4 tested systems.

The next graphs (Figure 5) show the purity of C-PC obtained in foamates and APC retained in retentates during the continuous FF processes.

**Figure 6:**
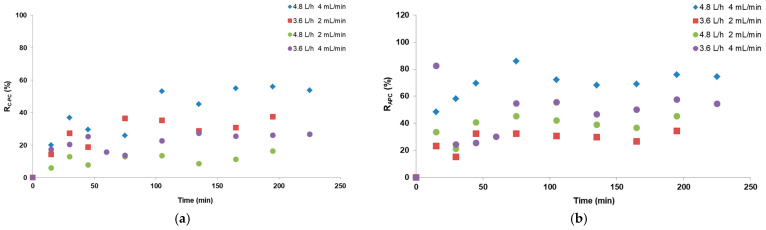
Recovery of (**a**) C-phycocyanin in the foamates and (**b**) allophycocyanin in the retentates in continuous foam fractionation for the 4 tested systems.

In processes 3–5, after 150 min from the beginning, the purities of C-PC in the foamates were at a similar level and amounted to approximately 1.3 (Figure 5a). The obtained values were similar to those obtained in the batch process at an air flow rate of 2.4 L/h except in process six (4.8 L/h and 4 mL/min), where the value of C-PC purity dropped to 0.9 after 200 min of the process. This was probably due to the most intensive mass transfer phenomenon, with a large amount of diluted foam flowing into the receiver. That is why in this variant, the highest C-PC recovery of approximately 60% was achieved while at the same air flow rate but with half as low flow rates on the pumps (experiment 5), the C-PC recovery in the condensate was the lowest and did not exceed 20%. These results are shown in the next graphs (Figure 6).

As is seen in the graphs (Figure 5b and Figure 6), during the continuous processes a partial separation of phycobiliproteins between the phases of foam condensates and retentates was achieved. The higher C-PC recovery in condensates and the higher APC recovery in retentates indicates the greater hydrophobicity of C-PC and its tendency to migrate to the interfacial surface. Conversely, APC remains in greater quantities in retentates, indicating its lower tendency for migration and concentration in the FF process. This was particularly visible in process no. 6 (with an air flow of 4.8 L/h and pumps of 4 mL/min), where the highest APC recovery in the collected retentate, reaching 90% (Figure 6b), was recorded. A similar phenomenon was observed in batch mode; however, the highest recovery of APC in retentate in that process was 60% (Figure 2b) and was achieved at an air flow rate of 2.4 L/h. To further compare, in continuous mode, at an air flow rate of 3.6 L/h, the average APC recoveries were 30% with pump flow rates of 2 mL/h and above 50% with 4 mL/h, while in the batch process for this flow rate, it reached 40% (Figure 2b).

**Figure 7:**
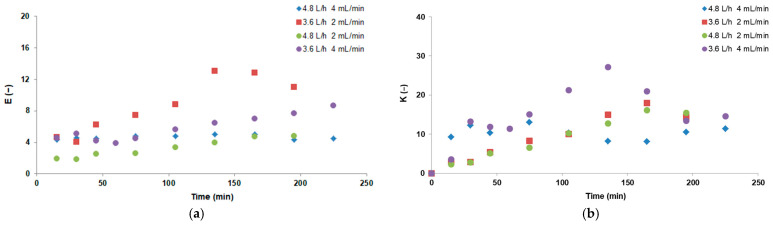
(**a**) Foam enrichment coefficients and (**b**) partitioning coefficients of C-phycocyanin obtained in continuous foam fractionation for the 4 tested systems.

In order to make a more comprehensive comparison, in the next graphs (Figure 7), the values of the foam enrichment coefficients and partitioning coefficients are presented.

The obtained results indicate that the calculated values of foam enrichment coefficient E (Figure 7a) were the highest in the processes with an air flow rate of 3.6 L/h, especially when the flow rate of 2 mL/min was set on the peristaltic pumps pressuring the crude extract and the retentate. In this variant, the residence time of the extract in the column and the liquid–gas contact were sufficient to obtain a condensate with a high C-PC concentration. The most significant increase in the E value occurred 130 min after the beginning of the continuous process. In the other processes, the foam enrichment coefficient did not change significantly over time. However, the average values of the partitioning coefficient K shown in Figure 7b were similar to those obtained in batch processes for the same air flow rates and ranged from 10 to 20 (Figure 3). The distribution of C-PC between the foamate and retentate changed slightly during the processes. Generally, the results indicate that foam fractionation can be applied on a larger scale, as the outcomes of the continuous processes were comparable to those of the batch ones. Similar results were obtained when continuous FF was investigated for the removal of per- and polyfluoroalkyl substances from landfill leachate [14].

## 3. Materials and Methods

### 3.1. Crude Extract of Phycobiliproteins

The crude extract of phycobiliproteins was obtained from the Department of Bioprocess Engineering as a product after bioreactor cultivation of *Synechococcus* sp. PCC 6715 described in Gluszcz et al. [21] and biomass disintegration and protein extraction described in Klepacz-Smółka et al. [22]. The average concentrations and purities of PBPs in the crude extract were as follows: 0.28 mg/mL and 1.0 for C-PC and 0.055 mg/mL and 0.44 for APC, respectively. It was stored in a freezer and used in ongoing experiments.

### 3.2. Foam Fractionation Process

#### 3.2.1. Experimental Set-Up

Foam fractionation of crude phycobiliprotein extract was conducted in the system presented in Blatkiewicz et al. [23]. A 62 cm long glass column, consisting of a working part, i.e., an inner tube with a diameter of 3 cm, was supplied with air at a pressure of 2 bar and an electronic flow rate regulator by Brooks Instrument (Hatfield, PA, USA). Air at the set flow rate was fed into the column through a silicone tube terminated with a glass air diffuser. At the top of the column, a semicircular tube for the foam, connected to a funnel with an internal diameter of 3 mm, was installed. After the foam was broken up into the form of condensate, it was collected in a Büchner vacuum flask equipped with a sample tube. A KNF Neuberger N022AN18 vacuum pump (KNF Neuberger GmbH, Freiburg, Germany) was used to generate underpressure.

**Scheme 1:**
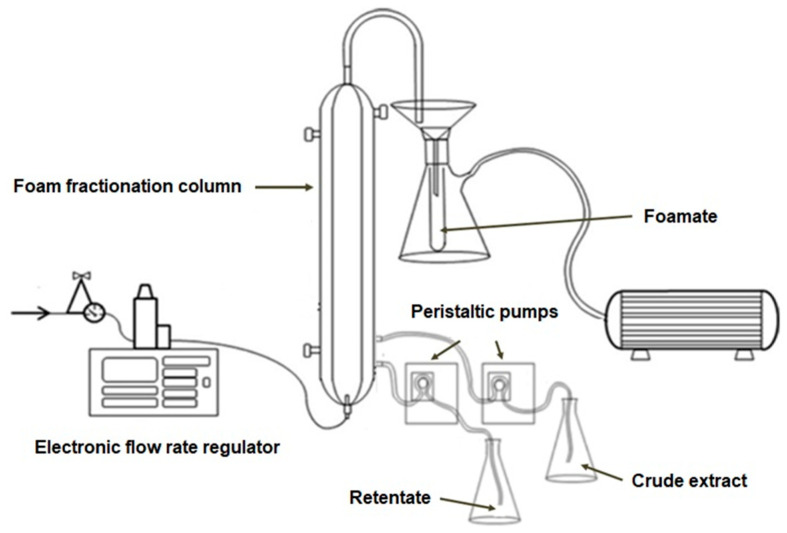
The scheme of the full experimental set-up for a foam fractionation process.

For continuous foam fractionation two Watson Marlow 101U/R peristaltic pumps (Watson-Marlow Limited, Cornwall, UK) were added. They were connected to the column with silicone tubes, which supplied the crude extract and simultaneously removed the excess retentate. The scheme of a full set-up is presented in the graph (Scheme 1).

#### 3.2.2. Running of the Process in Batch and Continuous Mode

Each process was started by filling the column with 100 mL of crude PBP extract and setting the air flow rate to 2.4 L/h. When the foam rose and reached the top of the column, the air flow rate was changed into the required value so during the process, a constant supply of air and underpressure in the receiver section were ensured. The process continued until the protein concentration was high enough to ensure the formation of stable foam, otherwise the process ended spontaneously. The obtained foam condensate (foamate), containing concentrated proteins, and the retentate remaining in the column were collected and analyzed. Based on the earlier experiments [15,16] all processes were conducted at room temperature (25 °C) and native pH (approximately 7.5). In batch mode, four different air flow rates (2.4; 3.5; 4.8; and 6.0 L/h) were investigated. As mentioned before, the continuous mode was carried out using two additional peristaltic pumps, one ensuring a constant supply of crude PBP extract and the second the collection of retentate to maintain a constant level of the fluid in the column. The retentate pump was switched on periodically. The process was started in the same way as in batch mode, but only once the foam reached the top of the column were the pumps started. The foamate and the retentate were collected periodically, every 15–30 min, their volumes were measured, and they were analyzed. Tests were carried out at three values of air flow rates, 2.4, 3.6, and 4.8 L/h, and two different flows set on the peristaltic pumps, 2 and 4 mL/min. Each process was run for 3 h, unless it was stopped earlier due to insufficient foam in the column.

### 3.3. Calculated Characteristic Values

The concentration of phycobiliproteins was determined spectrophotometrically using an EPOCH 2 microplate spectrophotometer (Agilent BioTek, Santa Clara, CA, USA) and then calculated using the following equations by Bennett and Bogorad [24]. All the spectrophotometric measurements of each sample were performed in triplicate.

(1) $$C_{C-PC}=\frac{A_{615}-{0.474\cdot A}_{652}}{5.34}\left[\frac{mg}{mL}\right]$$

(2) $$C_{APC}=\frac{A_{652}-{0.208\cdot A}_{615}}{5.09}\left[\frac{mg}{mL}\right]$$

where A is the absorbance in a given wavelength.

The purity of a given PBP indicates its quantity compared to other contaminating proteins and was calculated based on spectroscopic absorbance in two wavelengths as a ratio of absorbance in A_616_ for C-PC and A_652_ for APC to A_280_ [25].

The degree of volume reduction was calculated as the ratio of the crude extract volume used in the experiment to the volume of foam condensate formed after the process.

The purification factor (PF) indicating the increase in purity in a given process was calculated from the formula:

(3) $$PF=\frac{P_{p}}{P_{c}}$$

where P_p_ is the purity of a given PBP after the purification process and P_c_ is the purity of the PBP in the crude extract.

The recovery yield (R), which informs about the recovery of the C-PC or APC in a given method, was calculated from the formula:

(4) $$R=\frac{C_{p}\cdot V_{p}}{C_{c}\cdot V_{c}}\cdot 100\%$$

where C stands for concentration of C-PC/APC, V stands for volume, the index p refers to the phase where C-PC/APC is separated (the concentration of C-PC/APC is higher), and the index c refers to the crude extract introduced to the system.

The foam enrichment coefficient (E), calculated as the ratio of the C-PC concentration in the foamate to the C-PC concentration in the crude extract, is obtained as follows:

(5) $$E=\frac{C_{p}}{C_{c}}$$

The partitioning coefficient (K), which informs about the ratio of C-PC concentrations between the phases, is obtained as follows:

(6) $$K=\frac{C_{p}}{C_{s}}$$

where the index p refers to the phase with concentrated C-PC and the index s refers to the second phase of the analyzed system.

## 4. Conclusions

Foam fractionation can be successfully considered as an effective alternative method for purification of phycobiliproteins, especially phycocyanin and allophycocyanin, both in batch and continuous modes. The set-up from the batch mode experiments can easily be transformed with the use of two peristaltic pumps, one for the extract and the second for the removal of excess retentate, into a continuous one, ensuring better efficiency of the final product. The average recovery yields obtained are up to 60% with air flow rates from 2.4 to 4.8 L/h. A higher air flow rate of 6.0 L/h increases the recovery, but it also significantly reduces the partitioning and enrichment coefficients. Interestingly, for flow rates of 2.4–4.8 L/h, the separation of two phycobiliproteins between the foamate and retentate was observed. C-PC in greater quantities diffuses to the phase boundary, eventually forming condensate, and APC remains in the retentate. This means that the method gives the opportunity to separate phycobiliproteins from each other. The purity of the C-phycocyanin in the foamates from the batch process was about 1.3 and up to 1.5 from the continuous process, both allowing for its application as a food dye. Therefore, due to its advantages, simplicity, energy, and cost-effectiveness, the foam fractionation method is strongly recommended for further investigations and applications, especially as the first stage of the purification process.

## Figures and Tables

**Table 1:** Volumes of the three fractions from the foam fractionation (FF) process at different air flow rates.

Process No.	Air Flow Rate	Volume of Crude Extract	Volume of Foamate	Volume of Retentate	Degree of Volume Reduction
	[L/h]	[mL]	[mL]	[mL]	[-]
1	2.4	120	18	96	6.7
2	3.6	100	29	57	3.4
3	4.8	100	36	60	2.8
4	6.0	100	67	19	1.5

**Table 2:** Parameters for the foam fractionation processes carried out in the continuous mode.

Process No.	Air Flow Rate	Crude Extract and Retentate Flow Rate	Comment
	L/h	mL/min	
1	2.4	2	The process expired on its own
2	2.4	4	The process expired on its own
3	3.6	2	-
4	3.6	4	-
5	4.8	2	-
6	4.8	4	-

## Data Availability

All data generated or analyzed during this study are included in this published article or will be available on request.
